# A case report of encapsulating peritoneal sclerosis followed by cesarean section: Clinical diagnosis and treatment experience

**DOI:** 10.1097/MD.0000000000032122

**Published:** 2022-12-02

**Authors:** Jin Long Liang, Zheng Quan Chen, Zhang Yi, Wen Kun Ming

**Affiliations:** a Department of General Surgery, Digestive Disease Hospital, Affiliated Hospital of Zunyi Medical University, Zunyi, Guizhou, P.R. China; b Department of Gynecology of Zunyi first people’s Hospital, Zunyi, Guizhou, P.R. China.

**Keywords:** cesarean delivery, encapsulating peritoneal sclerosis, intestinal obstruction

## Abstract

Encapsulating peritoneal sclerosis (EPS), also known as abdominal cocoon syndrome, is an uncommon condition that typically presents with features of bowel obstruction. EPS followed by cesarean section is extremely rare. Intestinal obstruction caused by EPS lacks specificity and poses clinical difficulties for diagnosis and treatment. We present the case of a patient with recurrent intestinal obstruction followed by cesarean section, and the diagnosis of EPS was confirmed intraoperatively. The patient recovered well postoperatively, and achieved satisfactory therapeutic results. Management of this condition tests the surgeon’s knowledge and experience of the disease.

**Patient concerns:**

A 27-year-old woman with recurrent abdominal pain and distention accompanied by reduced anal discharge and defecation there months. The patient had a history of cesarean section 4 months earlier and recovered well after operation. She had no other history of abdominal surgery or diseases. On examination, a 10-cm long transverse incision was made in the lower abdomen, and marking on the intestinal movements were observed in the left mid-abdomen. A long, soft lump with good mobility was touched in the left lower abdomen. The abdominal computed tomography and small bowel barium meal examination revealed incomplete intestinal obstruction.

**Diagnosis:**

Incomplete small bowel obstruction due to abdominal adhesions after the cesarean section was initially considered.

**Interventions:**

After conservative treatment, the symptom of intestinal obstruction still recurred. Thus, we decided to perform a surgery of repeated decortication of fibrous peritoneal membranes.

**Outcomes:**

The operation successfully released the intestinal obstruction and abdominal pain, postoperative course recovered smoothly.

**Lessons:**

After cesarean section could develop EPS. Intestinal obstruction caused by EPS lacks specificity and poses clinical difficulty in diagnosis and treatment. The management of this condition tests the surgeon’s knowledge and experience, and surgery is an effective treatment measure.

## 1. Introduction

Encapsulating peritoneal sclerosis (EPS) is an extremely rare disease characterized by a dense, grayish-white fibrous membrane enveloping all or part of the small intestine. In 1978, Foo et al^[1]^ first used the term “peritoneal callus” to describe the condition. It is now commonly called as EPS,^[2]^ which describes the morphological and histological changes of the disease at the pathological level. EPS can be divided into primary and secondary. Primary EPS are also known as idiopathic EPS, which is the commonest to women from tropical and subtropical countries. It may be caused by gynecological infection and tubal infection or retrograde menstruation, which induces infectious peritonitis.^[3]^ Secondary EPS have many causes. It is commonly associated with a history of abdominal tuberculosis,^[4]^ chronic kidney disease, peritoneal dialysis,^[5]^ and so on. Due to the thickening of the peritoneum and restriction of bowel movement, EPS often present as a symptom of intestinal obstruction.^[6]^ In most cases, surgery remains the most effective treatment option.^[7]^ Here, we report the case of a patient with EPS followed by cesarean section who presented with recurrent episodes of intestinal obstruction. we operated on the patient and ultimately obtained a satisfactory result.

## 2. Case presentation

**Figure 1.**
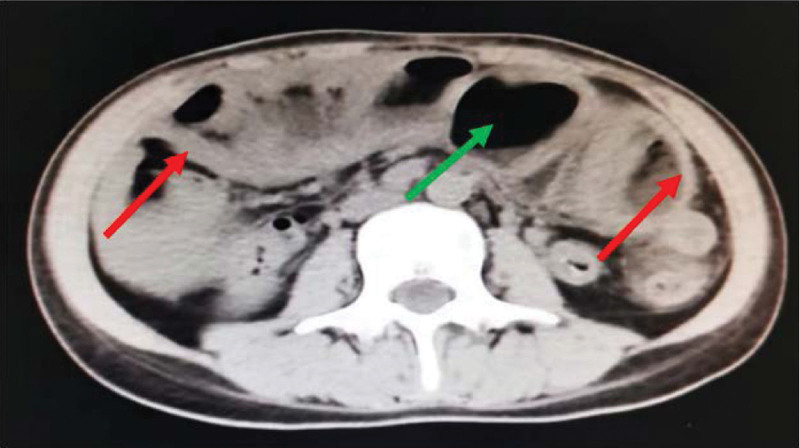
Multiple intestinal wall thickening (red arrow) and partial dilatation of the intestinal canal (green arrow).

**Figure 2.**
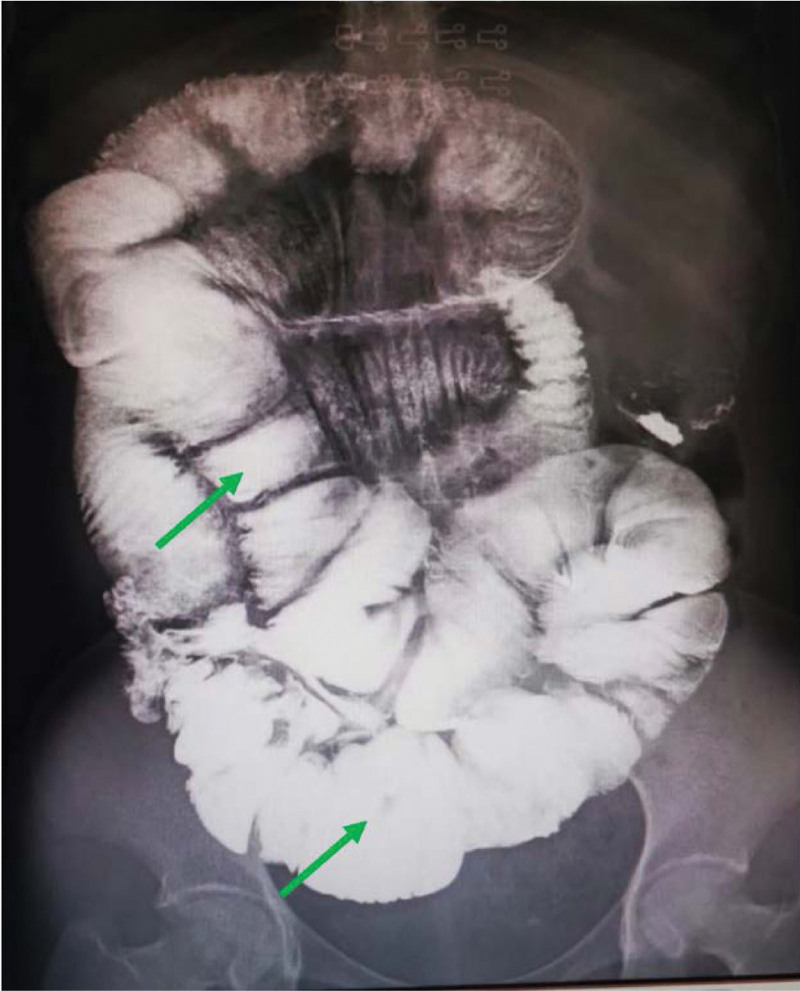
Enlarged loops of the distal ileum and ileocecum (green arrow).

A 27-year-old female patient presented to our institution with 3 months of abdominal pain and distension. She started to suffer paroxysmal abdominal pain and distension accompanied by reduced anal discharge and defecation 3 months earlier, which worsened after eating. The patient had undergone a cesarean section 4 months previously and recovered well after the operation. There is no other history of abdominal surgery or diseases. During this period, she had lost weight of 15 kg. After presented to several hospitals, she was diagnosed as incomplete intestinal obstruction. When the conservative treatment was conducted, the symptom of intestinal obstruction was released. However, the symptom was still recurrent when she discharged. Thereafter, the patient went back to our construction. Physical examination revealed that she was malnourished, and her body mass index was only 13.67 kg/m^2^. A 10-cm long transverse incision was observed on the lower abdomen, and intestinal movements was recurrently seen on the left mid-abdomen. A long, soft lump with good mobility was touched in the middle abdomen. No pressure pain, rebound pain and muscle tension in the whole abdomen; intestinal sounds were significantly reduced. Leukocytes: 7.54 × 10^9^/L neutrophil percentage 0.72; hemoglobin 126 g/L; albumin: 23.9 g/L; prealbumin 59 mg/L, both of albumin and prealbumin were significantly reduced. The abdominal computed tomography (CT) showed multiple intestinal wall thickening and partial of the intestinal canal was dilatated. (Fig. 1). Small bowel barium meal examination showed partial adhesions in the proximal jejunum, local intestinal stenosis, and incomplete intestinal obstruction in the distal ileum and ileocecal region, enlarged loops of the distal ileum and ileocecum can be seen (Fig. 2). Colonoscopy did not show any abnormality.

**Figure 3.**
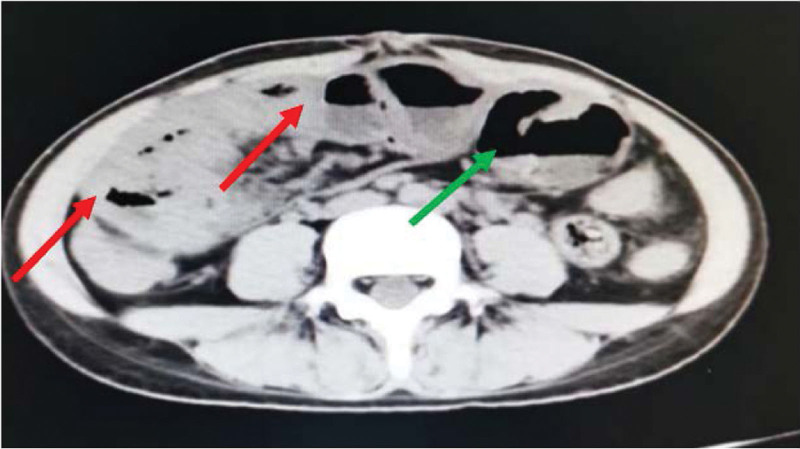
Multiple small intestinal wall thickening in lower abdomen (red arrow) and partial dilatation of the intestinal canal (green arrow).

**Figure 4.**
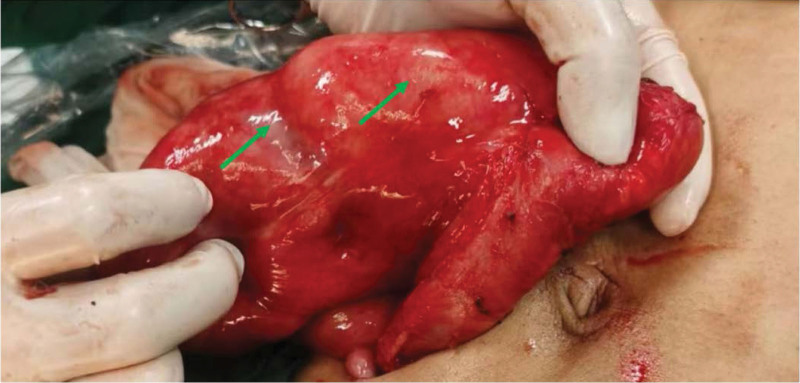
The surface of the small intestine and its mesentery is covered with a layer of fibrous membrane structure, and the small intestine is wrapped and confined (green arrow).

Incomplete Intestinal obstruction was considered and conservative treatment was given, including dietary abstinence, gastrointestinal decompression, enema, and complete parenteral nutrition support. Somatostatin maintained for 24 h, and slowly titrated to inhibit the secretion of digestive juices. A week later, the patient passed gas and stooled spontaneously. However, abdominal pain and distension were still existed after eating, the intestinal movement shape was still visible, and the long, soft lump in the left lower abdomen did not shrink and disappear. Repeat examination CT showed multiple small bowel wall thickenings in the lower abdomen, still suggesting inflammatory lesions and incomplete intestinal obstruction (Fig. 3). Blood routine: white blood cell 6.27 × 10^9^/L neutrophil percentage 0.50; hemoglobin 117 g/L; albumin: 36.7 g/L, prealbumin 214 mg/L. The patient’s state of malnutrition was significantly improved. Given this treatment result, we performed further surgical exploration. Intraoperatively, we found that the wall of the small intestine was thickened, shortening into lump, and the surface of the small intestine was covered with a membranous structure. The membrane was gradually thickened from the proximal to the distal end of the small intestine. The small intestine was enfolded and restricted its movement (Fig. 4). The surface membrane was carefully peeled off though operation, and the small intestine adhesions were decorticated, and normal peristalsis was restored. The proximal pole of the small intestine, about 60 cm long, which of the surface envelope was thin and difficult to separate. Thus, we resected this part, then the proximal and distal small intestine were anastomosed, and a drainage tube was placed near the anastomosis. The operation lasted 6 hours, and the patient was treated with complete parenteral nutritional support in the early postoperative period.

**Figure 5.**
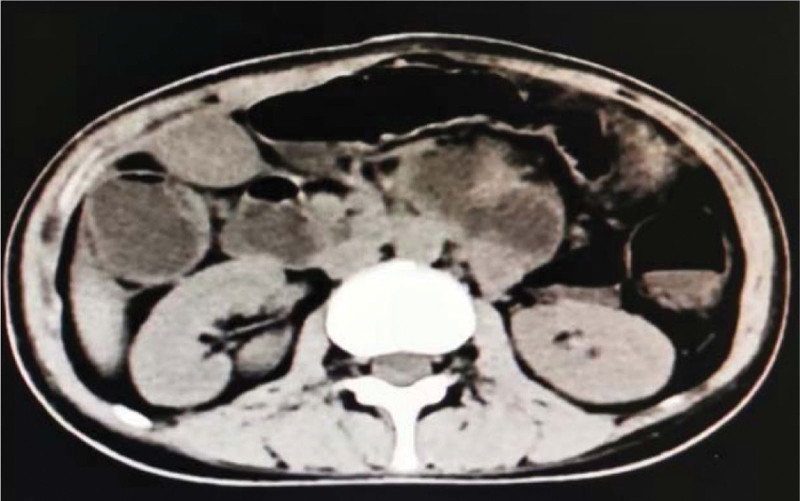
No liquid pneumoperitoneum sign in abdominal CT.

A week after the operation, the patient defecated, and bowel function was restored. Enteral nutritional preparations were increased, and the patient was instructed to start a liquid diet. However, after eating, there was a feeling of distension. So, tetracycline, morpholine, and bifidobacterial was given. somatostatin was maintained for 2 weeks to suppress digestive secretions after surgery. On the 12th postoperative day, a high fever with a temperature of 39°C was occurred. Even so, the patient had no signs of peritoneal irritation, and the routine blood test was repeated immediately: leukocytes 7.20 × 10^9^/L neutrophil percentage 0.76. There was no liquid pneumoperitoneum sign in the abdominal CT (Fig. 5). The possibility of an anastomotic fistula was ruled out, and then bacteremia due to central venous catheter infection was considered. Catheter tip culture and blood culture were performed, suggesting *Agrobacterium tumefaciens* and *Streptococcus pyogenes* infection. Anti-infective treatment with cefoperazone sulbactam combined with levofloxacin was selected according to the drug sensitivity results, and soon the infection was controlled. The patient was hospitalized for 25 days. The patient was followed up 1 month after discharge, her diet had returned to normal and with a good nutritional status.

### 2.1 . Postoperative pathology report

**Figure 6.**
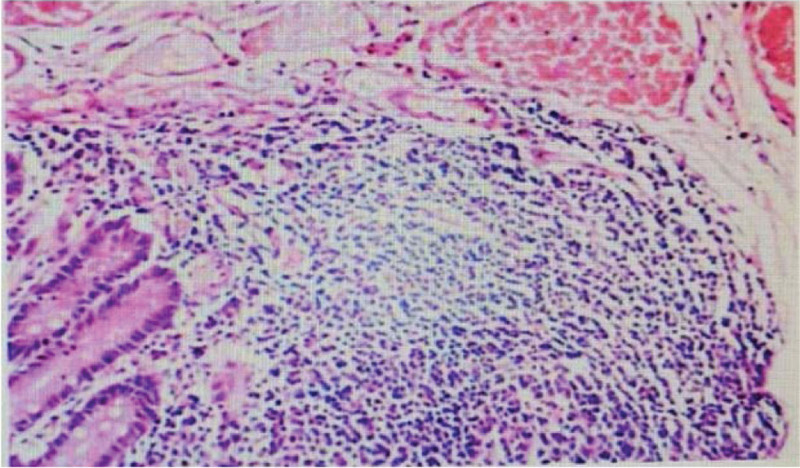
All layers of the intestinal canal were intact, and the submucosa was edematous, vasodilated, and congested with chronic inflammatory cell infiltration.

All layers of the intestinal canal were intact, and the submucosa was edematous, vasodilated, and congested with chronic inflammatory cell infiltration (Fig. 6).

## 3. Discussion

The etiology of EPS is diverse, and the pathogenesis is not yet clear. However, it is generally accepted that multiple associated triggers disrupt the normal physiological function of the peritoneum, causing an inflammatory response that leads to increased release of fibrogenic cytokines, resulting in fibrin deposition on the peritoneum.^[3]^ In this case, the thick peritoneum wraps around the intestine, leading to the adhesion of intestinal with each other, impeding intestinal peristalsis and eventually causing intestinal obstruction.^[6]^ There are many secondary causes of EPS, such as chronic peritoneal dialysis or abdominal trauma.^[8,9]^ However, EPS secondary to cesarean section has not been reported in the literature. This case reports a young female patient with EPS secondary by cesarean section, which severely affected the patient’s daily diet and led to significant weight loss and severe malnutrition. It is a rare condition. The cause of this EPS may be due to amniotic fluid or blood flows into the peritoneal cavity during cesarean section, which triggered an inflammatory response that led to peritoneal fibrosis and continuous thickening. Eventually, EPS was formed.

EPS is a rare cause of intestinal obstruction characterized by abdominal pain, distention, vomiting. Preoperative diagnosis is difficult due to a lack of knowledge and experience. As shown in the literature,^[10]^ abdominal CT scans are essential in the preoperative diagnosis of EPS. However, abdominal CT examinations lack specific signs, and diagnosis was mainly based on intraoperative observation.^[11,12]^ This suggests that the diagnostic significance of imaging seem to be low. In this case, preoperative abdominal CT revealed likely inflammatory lesions or incomplete bowel obstruction. Small bowel barium meal radiography showed partial adhesions in the proximal jejunum and local intestinal luminal stenosis and incomplete intestinal obstruction; both tests suggested the presence of incomplete small bowel obstruction. However, bowel obstruction caused by EPS was still not considered and diagnosed in the preoperative phase.

Whether surgical treatment should be conducted when it was uncertain that the intestinal obstruction was caused by EPS. In particular, the abdominal pain and distention were relieved, and the anus passed defecation, which were signs of incomplete intestinal obstruction. Although surgery is generally accepted as an effective treatment for EPS, patients with mild or asymptomatic intestinal obstruction can be treated conservatively with fasting, gastrointestinal decompression, and nasal-intestinal tube for parenteral nutritional support.^[13,14]^ After a week of conservative treatment, abdominal pain and distension were significantly released, which because of intermittently gas and stool passed from anal. It seemed possible to continue conservative treatment. However, the patient still complained of abdominal distension after eating and difficulty in anal venting and defecation. The bowel movement pattern was still observed, and a repeat abdominal CT still showed multiple small bowel wall thickening, partial intestinal dilatation, and incomplete intestinal obstruction; the abdominal lump was still touched. Therefore, we considered maybe it’s not a case of usual intestinal obstruction, we decided to perform surgery to explore with the patient’s approval. The intraoperative situation eventually proved the correctness and necessity of the surgical choice.

During the operation, we did not observe intestinal adhesions to the uterus or the original cesarean incision. We excluded intestinal obstruction caused by adhesions between the incision and the intestine after cesarean section, nor did we see adhesion band compression, etc. Instead, we found that the small intestinal wall was thickened, its length was shortened, and the surface was covered with an envelope, which wrapped the small intestine and the mesentery. Intestinal peristalsis was significantly restricted, and the diagnosis of EPS was confirmed intraoperatively.

The literature reports that surgical treatment of intestinal obstruction caused by EPS has definitive efficacy, releasing the encapsulated bowel by removing the fibrotic peritoneum without resecting the bowel is preferred.^[15]^ In this case, we carefully stripped the envelope from the surface of the distal small intestine and released the adhesions of the bowel. But, the superficial envelope of proximal small intestinal was too thin to be peeled off. Therefore, part of the proximal small intestine was removed, and then intestinal anastomosis was performed.

Anastomotic fistula is one of the serious complications after intestinal anastomosis. According to the literature,^[16]^ the most common risk factors for anastomotic fistula are pulmonary disease, previous albumin < 3.0 mg/dL, preoperative peritonitis, anastomotic strain, poor local blood supply to the anastomosis or infection, and presence of cancer and drainage placement. However, such as malnutrition, hypoproteinemia, and drainage placement were existed in this patient, which are the high-risk factor for intestinal fistula. Therefore, nutritional support throughout the entire treatment process, including enteral and parenteral nutritional support, early postoperative parenteral, intravenous nutrition, and gradually transition to an oral diet. Somatostatin effectively inhibit the release of gastrointestinal hormones and the exocrine function of the stomach to reduce motility of intestinal tract.^[17]^ In this case, considering that the anastomosis is close to the duodenum, and the risk of anastomotic fistula is highly likely, we indwelled a drainage tube near the anastomosis and used Somatostatin for 2 weeks to prevent the anastomotic fistula. Although infection symptoms occurred after operation, after eliminating anastomotic fistula, anti-infective treatment with sensitive antibiotics based on the drug sensitivity results, the patient recovered well and was discharged successfully.

## 4. Conclusion

In summary, EPS secondary to cesarean section is extremely rare, and the intestinal obstruction caused by EPS lacks specificity, making it difficult to diagnose and treat. The management of this condition tests the surgeon’s knowledge and experience. Surgery is an effective treatment option.

## Acknowledgments

We would like to thank the patient who consented to the publication of their cases in this paper.

## Authors contributions

JL Liang and KW Wen carried out the surgical intervention; ZC Chen collected, analyzed, and interpreted patients’ data. Y Zhang provided clinical care of this patient. All authors have read and approved the final version of the manuscript.

**Conceptualization:** JL Liang, KW Wen.

**Writing—original draft:** JL Liang.

**Writing—review & editing:** KW Wen.
